# Questioning the role of the frontopolar cortex in multi-component behavior – a TMS/EEG study

**DOI:** 10.1038/srep22317

**Published:** 2016-02-29

**Authors:** Krutika Gohil, Gabriel Dippel, Christian Beste

**Affiliations:** 1 Cognitive Neurophysiology, Department of Child and Adolescent Psychiatry, Faculty of Medicine of the TU Dresden, Germany

## Abstract

Cognitive control is central to many every day situations. There, we usually have to combine different actions to achieve a task goal. Several lines of research indicated that areas in the prefrontal cortex determine cognitive control in situations requiring multi-component behavior. One of this is the frontopolar cortex (FPC). However, direct non-correlative evidence for this notion is widely lacking. In the current study we test the importance of the FPC for the implementation of action cascading processes in a TMS/EEG study. The data, however, clearly show that the FPC does not modulate behavioral or neurophysiological parameters reflecting action cascading, which is in contrast to the findings of dorsolateral prefrontal cortex. The results are supported by a Bayesian analysis of the data. The results suggest that a possible role of the FPC in multi-component behavior needs to be refined. At least in situations, where multi-component behavior is achieved by stopping and switching processes and does not impose high demands on working memory processes the FPC seems to play no role in the implementation of this major cognitive control function.

Cognitive control processes are central to the organization of behavior. In most everyday situations behavioral control is complex, since most of our goals require subsequent execution of the different actions; i.e. multi-component behavior^1^. For this, we heavily depend on action cascading, which is defined as the ability to generate, process, and execute separate task goals and responses in an expedient temporal order to be able to display efficient goal-directed multi-component behavior^1^,^2^,^3^,^4^,^5^. For these processes it has been shown that the basal ganglia as well as prefrontal areas play an important role^2^,^6^,^7^,^8^,^9^. As concerns the prefrontal cortex, the right inferior frontal gyrus (rIFG) has recently been shown to causally determine action cascading processes^2^, especially when there is a choice when to process different response options and stopping and switching processes are involved. The rIFG is known to be part of the multiple demand (MD) system^1^ and it thus seems that the MD system is particular relevant for action cascading processes. However, as described above, successful action cascading depends on selecting subsequent actions based on information conveyed by past events and on the processing of subgoals during multitasking. These processes have also been referred to as ‘cognitive branching’ for which it has been suggested that the frontopolar cortex (FPC) plays an important role (e.g.^10^,^11^,^12^,^13^,^14^). The FPC is not part of the MD-system, but due to the above-mentioned properties may be relevant to the modulation of action cascading processes as well. In the current study, we investigate the role of the fronto-polar cortex for action cascading processes in a neuronavigated TMS/EEG study.

Multi-component behavior (action cascading) is examined using a stop-change paradigm (cf.^15^) in which participants are asked to occasionally stop a response and change to an alternative response. This change is either signaled at the same time with the stop signal, or shortly (300 ms) thereafter. Especially when there is a choice how to process stop and change stimuli (i.e. when both are presented at the same time) action cascading is particularly demanding. We apply continuous theta burst stimulation (cTBS) to modulate neural activity in the FPC and compared this to a sham TBS (sTBS) condition where the FPC is not stimulated. If the FPC plays a role in action cascading, modulations of the FPC will affect action cascading in situations when there is a choice how to process different response options. This is because in such situations demands on cognitive branching mechanisms are highest. As other neurophysiological studies have shown before, action cascading processes are reflected by modulations of the P3 event-related potential (ERP), which is well-known to reflect response selection and decision processes between stimulus evaluation and responding (e.g.^16^,^17^,^18^,^19^). However, the direction of a possible cTBS effect is unclear, as especially in the frontal cortex cTBS can have different effects^20^ sometimes also between homolog areas between hemispheres (e.g.^21^). In a recent study, we have shown that cTBS attenuates action cascading performance when applied to the right inferior frontal gyrus^2^. On the other hand, cTBS has been shown to diminish impulsive behavior and inhibitory control processes (e.g.^22^,^23^) playing a role in action cascading^2^. As inhibition processes play a central role in the paradigm applied to examine action cascading, it cannot be excluded that cTBS may also lead to improved action cascading processes.

## Results

### Behavioral data

The analysis of the reaction times (RTs) on GO trials revealed no main effect of TBS conditions on RTs (F(1,27) = 0.14; p = 0.71). A mixed effect ANOVA using the within-subject factors “SCD interval” and “TBS conditions” was run to analyze the RTs data on the CHANGE stimulus. There was a main effect of “SCD interval” (F(1,27) = 306.88; p < 001; η_p_^2^ = 0.919) indicating that the participants were generally slower in the SCD0 condition (906 ms ± 32) than the SCD300 condition (702 ms ± 34). However, there was no main effect of “TBS conditions” and no interaction of “SCD interval × TBS conditions” (F ≤ 0.13; p > 0.731). For the RTs in the SCD0 condition: cTBS = 913 ± 39; sTBS = 899 ± 33. For the RTs in the SCD300 condition: cTBS = 707 ± 40; sTBS = 690 ± 36. The analysis of SSRTs (mean SSRT = 254 ms ± 26.03) also showed no SSRT differences between TBS conditions (t(27) = −0.09; p = 0.923). Using bayesian statistics it is possible to provide a quantification of the degree to which the data supports the null hypothesis^24^ (i.e. no difference between TBS conditions). In other words; the probability of the null hypothesis being true, given the obtained data (*p*(H_0_|D)). Using the methods described by^24^ and^25^ we evaluate the strength of evidence that cTBS has no effect^3^. For all effects including the factor “TBS conditions” this analysis revealed *p*(H_0_|D) ≥ 0.90, thus providing strong evidence for the null hypothesis according to^26^.

A mixed effect ANOVA on the absolute frequency of correct responses on the CHANGE stimuli using the within-subject factors “SCD interval” and “TBS conditions” was run to analyze accuracy. There was a main effect of “SCD interval” (F(1,27) = 528.73; p < 0.001; η_p_^2^ =0.951) showing that participants were generally more accurate in the SCD300 condition (122.13 ± 1.71) than the SCD0 condition (90.59 ± 1.47). There was no main effect of “TBS conditions” (F(1,27) = 0.42; p = 0.519) and no interaction of “SCD interval × TBS conditions” (F(1,27) = 0.008; p = 0.928), indicating no differential effects of TBS conditions on both SCD intervals. For the accuracy in the SCD0 condition: cTBS = 91 ± 1.3; sTBS = 90 ± 2.1. For the accuracy in the SCD300 condition: cTBS = 122 ± 1.6; sTBS = 121 ± 2.1. This indicates that there were no differential effects of TBS conditions on RTs in the two SCD conditions. There was also no effect of TBS conditions on accuracy in GO trials (F(1,27) = 0.782; p = 0.607). For SC trials, the staircase procedure was applied to access SSRTs so the accuracy for the STOP response cannot differ. Similar to the RT data, the bayesian analysis showed that for all effects including the factor “TBS conditions”, the *p*(H_0_|D) ≥ 0.85, thus providing positive evidence for the null hypothesis^26^.

Summarizing the behavioral data, we only obtained known effects of SCD interval manipulation, but no effects of the cTBS protocol applied. The control region used for TBS stimulation, already shown to have no effects on performance in the task applied (cf.)^2^, also revealed no TBS effects: there were main effects of SCD interval, for both the RT and the accuracy data (all F > 56.45; p < 0.001). However, there was no difference between cTBS and sTBS in reaction times in the SCD0 (cTBS: 970 ± 20; sTBS: 955 ± 33) and SCD300 condition (cTBS: 980 ± 22; sTBS: 965 ± 40) (all F < 0.7; p > 0.6; *p*(H_0_|D) ≥ 0.90). The same was the case for the accuracy rates in the SCD0 (cTBS: 119.2 ± 1.74; sTBS: 117.8 ± 2.22) and SCD300 condition (cTBS: 93.34 ± 1.84; sTBS: 95.55 ± 3.01) (all F < 0.8; p > 0.6; *p*(H_0_|D) ≥ 0.90). This replicates previous findings (cf.)^2^.

### Neurophysiological data

#### Perceptual and attentional selection (P1 and N1 effects)

**Figure 1:**
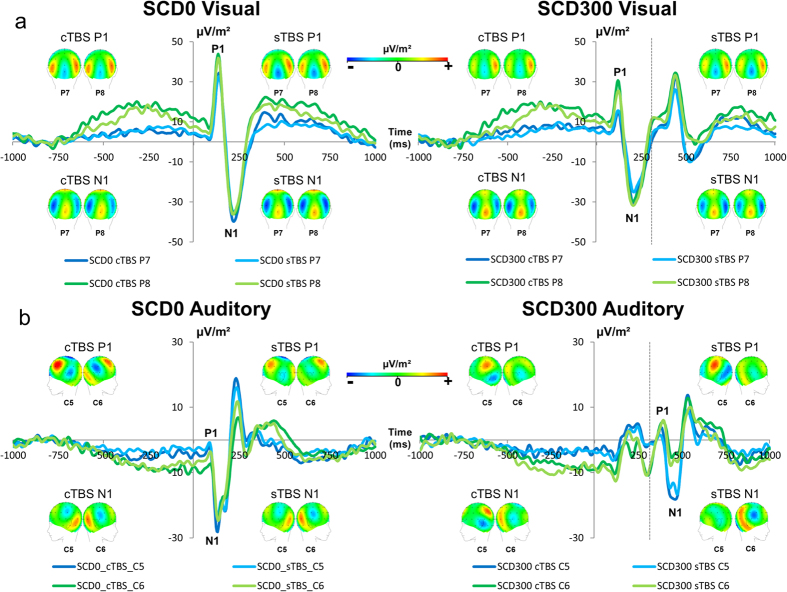
Illustration of the P1 and N1 ERPs in the SCD300 condition. Time point 0 denotes the STOP stimulus presentation and the vertical dashed line denotes the time point of the CHANGE stimulus presentation. All ERPs are shown for the cTBS and sTBS conditions. (**a**) Electrodes P7 and P8 show potentials upon the visual STOP stimulus for the SCD0 and the SCD300 conditions. (**b**) Electrodes C5 and C6 show potentials upon the auditory CHANGE stimulus for the SCD0 and the SCD300 conditions. The scalp topography plots show typical maps for the P1 and N1 ERPs.

All ERPs were quantified at the single subject level. Latencies are given relative to the onset of the stop signal (time point 0), and amplitudes were quantified relative to the pre-stimulus baseline. The ERPs on the P1 and N1 are shown in Fig. 1. The P1 ERPs were analyzed in a mixed effect ANOVA using the factors “SCD intervals,” “TBS conditions” and “electrode”, as within-subject factors for the visual STOP stimulus and the auditory CHANGE stimulus. For the visual P1, there was a main effect of “SCD intervals” (F(1,27) = 78.75; p < 0.001; η_p_^2^ = 0.745), showing that P1 amplitude was larger in the SCD0 condition (39.04 μV/m^2^ ± 2.92) than the SCD300 condition (23.072 μV/m^2^ ± 2.04). There was also a main effect of electrode (F(1,27) = 11.85; p = 0.002; η_p_^2^ = 0.305) indicating that the P1 was larger at electrode P8 (36.05 μV/m^2^ ± 3.196) than at electrode P7 (26.05 μV/m^2^ ± 2.25). However, there was no main effect of TBS conditions (F < 0.30; p > 0.6) and no other interaction effects (all F ≤ 0.74; p ≥ 0.5). For the auditory P1, there were no main effects or interactions (all F ≤ 0.94; p ≥ 0.4). The bayesian analysis for all effects including the factor “TBS conditions” showed that for all effects including the factor “TBS conditions”, the *p*(H_0_|D) ≥ 0.87, thus providing positive evidence for the null hypothesis^26^.

The N1 ERP was analyzed with the same kind of mixed effects ANOVA as the P1. For the visual N1, there was a main effect of “SCD intervals” (F(1,27) = 41.42; p < 0.001; η_p_^2^ = 0.605) indicating that N1 amplitude was larger (more negative) in the SCD0 condition (−46.38 μV/m^2^ ± 3.67) than the SCD300 condition (−36.87 μV/m^2^ ± 4.05). There was also a main effect of electrodes (F(1,27) = 5.08; p = 0.032; η_p_^2^ = 0.159), showing that the N1 was larger at electrode P8 (−45.83 μV/m^2^ ± 4.85) than at electrode P7 (−37.41 μV/m^2^ ± 3.50). There was no main effect of “TBS conditions” (F(1,27) = 2.51; p = 0.119). There were no other interactions (all F ≤ 0.49; p ≥ 0.413).

For the auditory N1, there was a main effect of SCD intervals (F(1,27) = 109.99; p < 0.001; η_p_^2^ = 0.803) indicating that the N1 amplitude was larger (more negative) in the SCD300 condition (−19.62 μV/m^2^ ± 2.13) than in the SCD0 condition (−5.44 μV/m^2^ ± 1.363). There was a main effect of electrodes (F(1,27) = 15.45; p = 0.001; η_p_^2^ = 0.364), showing that the N1 amplitude was larger at electrode C5 (−15.61 μV/m^2^ ± 1.77) than at electrode C6 (−9.46 μV/m^2^ ± 1.88). There were no main effects of TBS conditions (all F < 0.36; p > 0.7). There was an interaction of SCD intervals × electrodes (F(1,27) = 8.73; p = 0.006; η_p_^2^ = 0.244) and further Paired Samples Post-hoc tests revealed that the C5 differed from the C6 electrode on SCD300 trials (t(27) = −4.13; p < 0.001), but not on SCD0 trials (t(27) = −1.47; p = 0.151). There were no other interactions (all F ≤ 0.40; p ≥ 0.6). As with the P1 data, a bayesian analysis was run for the N1 data. The bayesian analysis for all effects including the factor “TBS conditions” showed that for all effects including the factor “TBS conditions”, the *p*(H_0_|D) ≥ 0.90, thus providing positive evidence for the null hypothesis^26^. Summing up the findings of attention related ERP components; there were no TBS-related effects, similar to the effects observed in the behavioral data.

#### Response selection processes (P3 effects)

**Figure 2:**
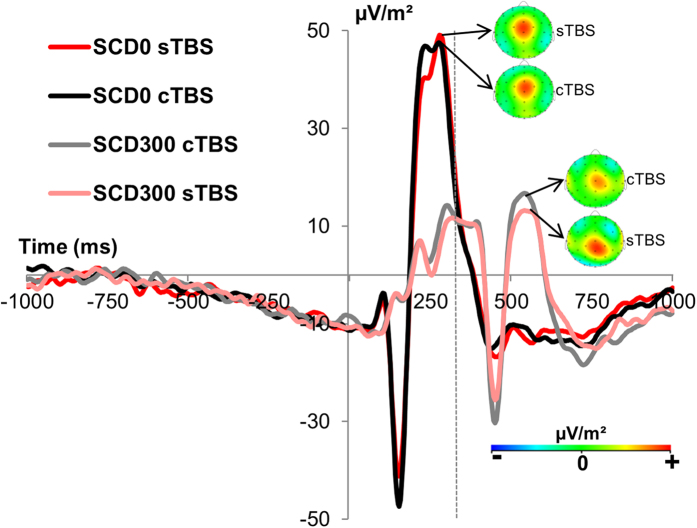
Illustration of the P3 ERPs. Time point 0 denotes the STOP stimulus presentation and the vertical dashed line denotes the time point of the CHANGE stimulus presentation. The cTBS and sTBS conditions are shown in the SCD0 and in the SCD300 condition. All ERPs are shown for electrode Cz due to the scalp topography. The scalp topography plots show typical maps for the P3 ERPs in the paradigm applied.

All ERPs were quantified at the single subject level. Response selection processes were examined by means of the P3 at electrode Cz (refer Fig. 2). The mixed effects ANOVA using the factors “SCD interval” and “TBS conditions”, and as within-subject factors revealed a main effect of “SCD interval” (F(1,27) = 168.68; p < 0.001; η_p_^2^ = 0.862), showing that the P3 amplitude was larger in the SCD0 (64.29 μV/m^2^ ± 4.52) than in the SCD300 condition (22.72 μV/m^2^ ± 2.62). There was no main effect of TBS conditions (F(1,27) = 0.12; p = 0.726). There was no interaction of “SCD interval × TBS conditions” (F(1,27) = 0.008; p = 0.93) indicating no differential effects of TBS conditions on both SCD intervals. Again, the bayesian analysis for all effects including the factor “TBS conditions” showed that for all effects including the factor “TBS conditions”, the *p*(H_0_|D) ≥ 0.95, thus providing strong evidence for the null hypothesis^26^. Summing up the findings of P3 ERP component, there were no TBS effects on SCD intervals and this again parallels the pattern found for the behavioral data.

## Discussion

In the current study we examined the importance of the frontopolar cortex (FPC) for action cascading processes using continuous theta burst stimulation (cTBS). The results show that the FPC plays no causal role in modulating action cascading processes. Neither behavioral parameters, nor any neurophysiological parameter was modulated as a function of cTBS applied to the FPC. This was supported by a bayesian analysis of the data. The results are unlikely to be explainable by an insufficient stimulation protocol because the same protocol has been shown to produce robust effects in other prefrontal brain areas (cf.)^2^ including the FPC^27^.

Previous results suggest that the FPC plays an important role in cognitive branching processes (e.g.^11^,^12^,^13^,^14^,^28^). In conceptions of the FPC it is assumed that the FPC is involved in multitasking behaviors^10^, especially when subjects postpone one task to perform the other^28^. The FPC has been suggested to enable a consideration of multiple task sets and goals^10^. This is, however, needed in the SCD0 condition, where STOP and CHANGE stimuli are presented together and thus activate STOP and CHANGE task goals at the same time^15^. In this condition it is therefore necessary to postpone processes related to the CHANGE stimulus until the stop process is finished, or try to process both aspects at the same time (e.g.^2^,^3^,^15^). In the SCD300 this is not the case, as a clear temporal structure is given. In the SCD0 condition, cognitive operations thus require simultaneous engagement in multiple tasks, a function that has been attributed to the FPC^10^. Yet, no modulation of behavior and neurophysiological processes by manipulating the FPC is observed. The results therefore question the importance of the FPC in “multi-tasking” or “multi-component behavior”. There are several possible explanations why the FPC does not modulate action cascading processes, as examined in the task applied:

As shown previously, the neurophysiological data revealed modulations of the P3 amplitude, which was larger in the SCD0 than in the SCD300 condition (cf.)^29^,^30^. These modulations are likely to reflect processes at the strategic central response selection bottleneck (e.g.^31^,^32^), i.e. processes reflecting the link between stimulus processing and the response (e.g.^16^,^17^,^18^,^19^). However, for such selection processes between task goals governing ongoing actions, it has been suggested that lateral prefrontal regions are important^10^. Concerning the lateral prefrontal cortex (LPC), it has been suggested that these regions, in particular the rIFG, causally modulate performance and neurophysiological processes during action cascading in the same task applied in the current study^2^. Opposed to the LPC, the FPC may only maintain a previously selected task set in a pending state to execute this action after another action has been completed^10^, but is not involved in the selection process per se.

Another critical factor, related to the above, may be the timing in the activation of task goals. In the SCD0 condition the STOP and the CHANGE task goal are simultaneously activated, so different task goals are selected during ongoing actions. This fits to the supposed role of the LPC^10^. However, in the paradigm applied the two required actions (i.e. stopping and changing) can be processed in rapid succession. Therefore, demands to integrate short-term representations over time are rather low: First, because the time duration is only short, and second because not many task goals have to be held online. It is possible that this lack of a strong working memory component in the task applied to examine multi-component behavior explains why cTBS on the FPC does not modulate behavior. This fits with findings underlining the particular importance of the FPC (BA10) for working memory processes (e.g.^33^,^34^).

A last factor why there was no effect of FPC on action cascading may relate to importance of stopping processes in the task applied. Results from lesion mapping have shown that FPC is not involved in cognitive control processes (response inhibition, conflict monitoring, and switching), but more involved in value-based decision-making^35^. The latter processes are not important in the task applied.

Together the results suggest that the proposed role of the FPC in multi-tasking or multi-component behavior needs to be refined. At least in situations, where multi-component behavior is achieved by stopping and switching processes and does not impose high demands on working memory processes the FPC seems to play no role in the implementation of this major cognitive control function.

## Materials and Methods

### Participants and study design

A sample of n = 28 right-handed participants; (11 males), 25 ± 2.38 years of age was examined. All subjects were healthy and conformed to TMS safety criteria^36^,^37^. All subjects gave written informed consent. The study was approved by the ethics committee of the medical faculty of the Technische Universität Dresden. The methods were carried out in accordance with the approved guidelines. There were three appointments: The first session served to obtain the participants’ individual structural images. In sessions two and three, an EEG was recorded while participants performed the action cascading task. This was done immediately after either continuous theta burst stimulation (cTBS) or sham theta burst stimulation (sTBS) was applied (details see below). The sequence of TBS protocols was counterbalanced across the subjects. To control for the effects of cTBS in the FPC, a group of n = 10 subjects was recruited undergoing cTBS in the middle frontal gyrus (details see further below).

### Task

**Figure 3:**
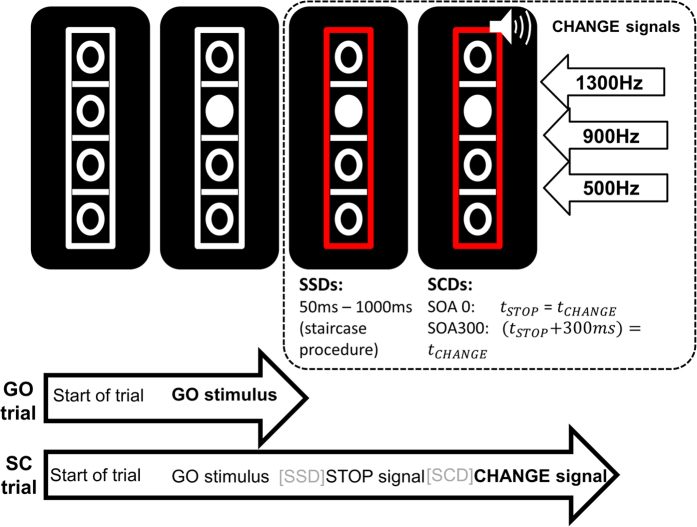
Illustration of the stop-change task (SCT) to examined action cascading. The experiment presented a visual GO signal at the beginning of all trials. In GO trials, the subjects needed to respond with the right hand. In stop-change trials, the GO stimulus was followed by a visual STOP stimulus (red rectangle) after a variable and individually adjusted stop-signal delay (SSD). The auditory CHANGE stimulus was either presented with a stop-change delay (SCD) of 0 ms or of 300 ms. Responses to the CHANGE stimulus had to be given with the left hand.

We used the same paradigm as in a previous TMS study by our group (cf.)^2^, a modified paradigm introduced by Verbruggen^15^ (refer Fig. 3). All participants were seated at a distance of 57 cm from a 17 inch CRT computer monitor in a quiet room. Presentation software (Version 17.1 by Neurobehavioural Systems, Inc.) was used to present the stimuli, record the behavioural responses (Reaction times (RTs) and correct responses) and to synchronize with the EEG. The participants were instructed to respond using four different keys (“1”, “2”, “3”, and “4”) located on a customized computer keyboard placed in front of them. The task consisted of 66 percent of “GO” trials and 33 percent of “Stop-Change” (SC) trials (864 trials in total), presented in a pseudorandomized order. The stimuli consisted of 4 vertically arranged white bordered circles, which were separated by 3 white horizontal lines. This stimulus array was enclosed in a white bordered rectangle (see Fig. 3). Each trial began with an empty array of these stimuli. One of the four circles was filled with white color after 250 ms and this white circle became the target (GO stimulus). Participants were asked to respond to this target by pressing either “1” or “2” keys with the right hand. In case, the target was located above the middle white line (i.e., the reference line) participants had to respond with their right middle finger (by pressing “1” key). In case the target was located below the middle line, participants had to respond with their right index finger (by pressing “2” key) for the correct key response. A speed up sign (the German word “Schneller!” which translates to “Faster!”) was presented above the stimulus array, if participants did not respond within 1000 ms after the onset of the target. The sign remained on the screen until the trial was ended.

On SC trials, the GO stimulus was followed by a STOP stimulus after a variable Stop-signal delay (SSD). As a STOP stimulus, the border of the rectangle turned from white to red (refer Fig. 3). The SSD was adapted to each participant’s individual task performance by means of a staircase algorithm^15^,^38^. Initially, the SSD was set to 250 ms. If the participant was able to refrain from responding on the GO stimulus upon presentation of the STOP stimulus, the SSD was decreased by 50 ms. If the participant responded on the GO stimulus, the SSD was increased by 50 ms. This staircase procedure yielded a 50% probability of successfully performed SC trials. SSD variation was restricted to a range from 50 to 1000 ms. The STOP stimulus was always followed by a CHANGE stimulus and required participants to respond with their left hand fingers (“3” and “4” keys). There were two CHANGE conditions: In the first condition, there was no delay between the STOP and the CHANGE stimulus (SCD0). In the second condition, the CHANGE stimulus was presented 300 ms after the onset of the STOP stimulus (SCD300). Our experiment used an auditory CHANGE stimulus, which was a 200 ms long sine tone presented via headphones. There were three pitches presented at a 75dB sound pressure level (i.e. low/600 Hz, middle/900 Hz, and high/1200 Hz), which were indicative of one of the three lines. The middle tone represented the middle reference line while the high and low tones stood for the high and low reference lines, respectively. Upon presentation of the CHANGE stimulus, participants were asked to spatially relate the target (white circle) to the new reference line that was indicated by the CHANGE stimulus. In case, the target was located above this new reference line (indicated by the CHANGE stimulus), participants had to respond with their left middle finger (by pressing “3” key). If the target was located below the new reference line, then participants had to respond with their left index finger (by pressing “4” key). In case participants did not respond within 2000 ms after the onset of the CHANGE stimulus, the speed up sign was presented.

### Continuous theta burst stimulation procedure

**Figure 4:**
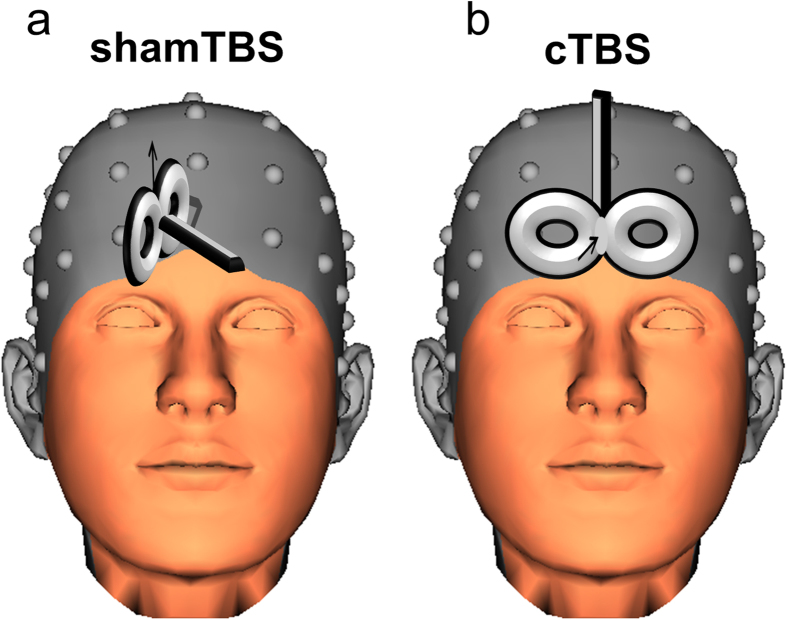
Graphical illustration of the TMS coil. The head was taken from the BrainVision Analyzer 2.1 software package (http://www.brainproducts.com/productdetails.php?id=17) and it shows the used electroencephalogram electrode grid. Both of the figures show the direction of the magnetic field by the black arrow. Contrary to the cTBS condition (right Figure part b), in the sham condition the coil is orientated at the 90 degree angle and a piece of rubber was placed between the TMS coil and head, so the magnetic field or any residual magnetic influences do not affect the frontopolar cortex(left Figure part a).

**Figure 5:**
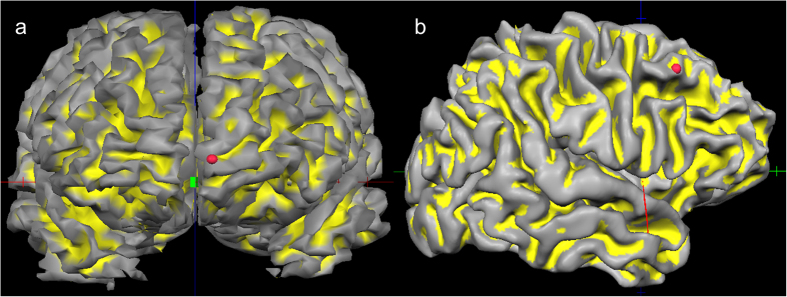
Illustration of the localization of the TBS stimulation area. Both figures show the localization of the stimulation area and the red point denotes the point of stimulation in the frontopolar cortex (FPC) (Figure part a) and the control region in the middle frontal gyrus (Figure part b).

The theta burst stimulation protocol was identical to our previous study (cf.)^2^. Transcranial magnetic stimulation (TMS) was applied using a PowerMAG research 100 device (MAG & More GmbH, DE) (double coil: 196 mm × 100 mm × 13.5 mm). At first, using established protocols (cf.)^39^ the resting motor threshold (RMT) was determined by means of electromyography (EMG). The wire electrodes were placed on the skin over the target adductor pollicis muscle and **s**ingle TMS pulses were applied to the primary motor cortex’s hand area (left M1). The stimulation intensity was increased in steps of 10% of the device’s maximum stimulator output until the first motor evoked potential (MEP) was identified. The precise RMT was assessed by sequentially increasing the intensity until five out of ten MEPs (peak-to-peak) of at least 50 μV were registered. In line with current safety standards^40^ continuous TBS (cTBS) was applied according to the protocol by^41^. The TBS consisted of bursts containing 3 pulses of 50 Hz at an intensity of 70% RMT repeated at 200 ms intervals (i.e., at 5 Hz frequency). During each TBS session, 600 pulses were applied. cTBS consisted of 40 s of continuous TBS. For sham stimulation, the coil was tilted away from the scalp in a 90° angle, and a rubber spacer was placed between the subject’s head and the coil (as shown in Fig. 4a). Accurate targeting was confirmed throughout the TBS stimulation using the neuronavigation system. For this, we first obtained 3D T1-weighted anatomical brain images on a 3 Tesla HDxt whole-body MRI scanner (GE Signa) using an 8-channel head coil (BRAVO 8 channel brain) with magnetisation-prepared gradient echo images (TR: 10.36 ms; TE: 4.2 ms; FA: 13°; 160 sagittal slices; matrix size, 512 × 512; FOV, 240 × 216 mm; 0.5 × 0.4 × 1.2 mm voxels). Neuronavigation was performed using the BrainVoyager TMS Neuronavigation System (BrainInnovation, Maastricht, The Netherlands) with established guidelines^42^. As target coordinate we choose ([x, y, z] = [−6, 60, 10]) (as shown in Fig. 5a) and targeted this with the TMS coil (as shown in Fig. 4b). based on a previous EEG-source localization study on action cascading using the same paradigm providing evidence for the involvement of the FPC together with activation differences in the inferior frontal cortex (cf.)^43^. As a control region, a region in the middle frontal gyrus was used ([x, y, z] = [23, 29, 41]) (cf. Fig. 5b). This control region is the same as used in a previous study on action cascading (cf.)^2^.

### EEG recording and analysis

The EEG was recorded 60 TMS-compatible electrodes (sampling rate 5 kHz; electrode impedances < 5 kΩ) connected to a BrainAmp DC amplifier (BrainProducts, Inc.)^2^. (reference electrode: Fpz). Data analysis was performed using the Brain Vision Analyzer 2 software package. Offline, the EEG was down-sampled to 256 Hz and filtered using an IIR filter (0.5–20 Hz, at 48 db/oct each) before gross technical artifacts were removed during visual inspection of the data. The used software automatically applies the appropriate filter before downsampling. We added this to the methods section to ensure the respect of the Nyquist theorem. Eye blinks, saccades and pulse artefacts, were corrected using an independent component analysis. Next, stimulus-locked segments were formed on the presentation of the STOP stimulus. Segments started −2000 ms before and ended 2000 ms after STOP stimulus presentation. After segmentation, an automated artefact rejection procedure was applied (rejection criteria: maximum voltage step of more than 60 μV/ms, maximal value difference of 150 μV in a 250-ms interval, activity below 1 μV). Artefact rejection was followed by a current source density (CSD) transformation, yielding a reference-free evaluation of the electrophysiological data which helps to identify electrodes showing the strongest effects^44^. Baseline correction was conducted using the interval from −900 ms to −700 ms as the pre-stimulus baseline (i.e., a baseline set prior to the occurrence of the GO stimulus). Based on the scalp topography maps, the visual P1 and N1 were quantified at electrodes P7 and P8 (P1: 25 ms until 85 ms; N1: 90 until 190), the auditory P1 and N1 were quantified at electrodes C5 and C6 for the SCD0 condition and SCD300 condition (SCD0 P1: 25 ms until 85 ms; N1: 90 until 190; SCD300 P1: 330 ms until 480 ms; N1: 390 until 490), and the P3 was quantified at electrode Cz (SCD0: 190 ms until 430 ms; SCD300: 190 ms until 740). The choice of electrode positions for data quantification was statistically validated as described in^17^.

## Data analysis

The data were analysed using repeated measures ANOVAs with the within-subject factors “SCD interval” (SCD0 vs. SCD300), “condition” (TBS protocols), and “electrode” (wherever necessary). Greenhouse-Geisser correction was applied and post-hoc tests were Bonferroni-corrected whenever necessary. All included variables were normally distributed as tested with Kolmogorov-Smirnov tests (all z < 0.5; p > 0.6). The means and standard error of the mean (SEM) are given.

## Additional Information

**How to cite this article**: Gohil, K. *et al*. Questioning the role of the frontopolar cortex in multi-component behavior - an TMS/EEG study. *Sci. Rep*. **6**, 22317; doi: 10.1038/srep22317 (2016).
